# Momentary PERMA: An Adapted Measurement Tool for Studying Well-Being in Daily Life

**DOI:** 10.1007/s10902-023-00684-w

**Published:** 2023-09-22

**Authors:** Saeideh Heshmati, Nermin Kibrislioglu Uysal, Sharon H. Kim, Zita Oravecz, Stewart I. Donaldson

**Affiliations:** 1 Department of Psychology, Claremont Graduate University, Claremont, CA USA; 2 Intersystems Corporation, Cambridge, USA; 3 Human Development and Family Studies, Pennsylvania State University, University Park, USA

**Keywords:** PERMA, Well-being, Intra-individual variability, Dynamical characteristics, Ecological momentary assessment, Experience sampling, Daily life

## Abstract

PERMA is a multidimensional framework that explains well-being through five hedonic and eudaimonic psychological elements–Positive emotions, Engagement, Relationships, Meaning, Accomplishment. Soon after the PERMA framework was proposed, PERMA-Profiler was introduced as a validated assessment tool for measuring these five elements of well-being from a global perspective. The current study aimed to shed further light onto the measurement of PERMA elements, extending it beyond global evaluations, to daily life assessments and the examination of individual differences in their dynamic characteristics. We introduce *m*PERMA (*momentary* PERMA), as an EMA-adapted version of the PERMA-Profiler measure, to assess well-being in daily life. Using data collected in an Ecological Momentary Assessment (EMA) study (N = 160), we first demonstrate the factor structure of *m*PERMA through a multilevel factor analysis and next examine within-person means and the dynamics of change (e.g., intra-individual variability) in the PERMA elements. Findings revealed that *m*PERMA displays convergent validity with two global measures of hedonic and eudaimonic well-being, namely Flourishing and Subjective Well-Being. Moreover, dynamical characteristics of the five elements of well-being measured over time, map onto their corresponding hedonic or eudaimonic global measures of well-being. Results of this paper present how dynamical features of well-being in daily life provide novel insights into predicting global well-being.

**Supplementary Information:**

The online version contains supplementary material available at 10.1007/s10902-023-00684-w.

Positive Psychology, as a new field of study (Seligman & Csikszentmihalyi, 2000), has brought about a wave of scientific investigations around the understanding of well-being and the good life. One recent approach to understanding well-being has been the building blocks of PERMA, which has been studied and discussed extensively in the *Journal of Positive Psychology* (e.g., Donaldson, et al., 2020; Goodman, et al., 2018; Kern, et al., 2015; merritt et al., 2023; Seligman, 2018). In the field of positive psychology, well-being is generally viewed as containing two dimensions: hedonic well-being (i.e., seeking pleasure and enjoyment) and eudaimonic well-being (i.e., virtuous living and functioning well). Whereas some theories of well-being approach the examination of well-being from one of these dimensions, the PERMA theoretical perspective views well-being as a multidimensional construct containing both hedonic and eudaimonic dimensions. PERMA explains well-being through five elements–some pertaining to the hedonic dimension and some to the eudaimonic– that all together contribute to individuals’ experiences of well-being (Seligman, 2011). The five elements of PERMA are:

Positive emotions: the hedonic experiences of positivity, contentment, and pleasure,

Engagement: the experience of being in flow while losing track of time,

Relationship: experiencing cared for and loved by others,

Meaning: having a sense of purpose in life, and

Accomplishment: the successes and achievements experienced in life.

While this theory incorporates certain elements of hedonic and eudaimonic well-being, it is important to recognize that it lacks certain elements found in other prominent theories of psychological well-being (e.g., personal growth and self-acceptance in Ryff’s (1989) theory of psychological well-being). A recent debate has transpired in the field around the redundancy of PERMA as a new conceptual framework of well-being and whether it is sufficiently distinct from previous well-being frameworks. In a comparison study of PERMA and Subjective Well-Being (SWB; Diener, 1984), Goodman et al. (2018) concluded that measures of PERMA and SWB were so highly correlated (*r* = 0.98) that they “represent the same type of well-being” (p. 327) with further evidence from an Exploratory Structural Equation Modeling showing “considerable overlap between the two well-being models” (P. 328). Goodman et al. (2018) hence argue that while the facets described within each framework are different, they both capture the same “type” of well-being. In response to Goodman et al. (2018), Seligman (2018) argued that Goodman and colleagues’ (2018) findings were in-line with his own hypotheses: PERMA captures the same type of well-being as SWB. However, Seligman (2018) posited that Goodman et al.’s (2018) “conclusion that PERMA is redundant with SWB and theoretically arbitrary is [in his belief] incorrect” (Seligman, 2018; P. 1) and that “Goodman et al.’s (2018) data exactly confirm that PERMA constitutes (at least some of the) elements of well-being” and that these building blocks are interrelated in nature (cf. Heshmati et al., 2020). To further examine the soundness of the PERMA framework and PERMA-Profiler (Butler & Kern, 2016) as its measure of well-being, subsequently, through a multi-trait multi-method (MTMM) research design, Donaldson et al. (2021) addressed the mono-method and self-report biases in the measurement of well-being in relation to SWB and PERMA. This research examined both people’s self-reports as well as their colleagues’ reports (collateral) of their PERMA well-being indicators in relation to their SWB. Donaldson and his colleagues (2020) found that even beyond the sole use of self-report measurement, PERMA and four additional building blocks (physical health, mindset, physical work environment, economic security) through collateral reports are significantly predictive of SWB and thus constitute a sound framework of well-being.

Although previous empirical studies have addressed some of the confusion around the validity and reliability of measures of PERMA, in particular in relation to SWB, we still lack understanding on how the PERMA building blocks relate to well-being in *daily* life. Namely, we do not know whether PERMA elements, as experienced in daily life, are distinct enough to be measured in a multidimensional manner rather than being examined as a global assessment of well-being.

## Well-Being Theories and Applications in Real-World Settings

At the heart of most operationalizations of well-being lies a distinction between two fundamental components of well-being: hedonia and eudaimonia. Hedonia is the experience of positive emotions or the “good feeling” (Henderson, et al., 2014). Eudaimonia refers to virtuous living and “functioning well” (Boehm et al., 2016). By definition, hedonia and eudaimonia are interrelated where feeling good can be experienced in tandem with functioning well.

In the field of positive psychology, some frameworks defining well-being have only targeted one component of well-being, either hedonic well-being or eudaimonic well-being. For example, Ryff’s theory of Psychological Well-Being (Ryff, 1989) is a multifaceted framework of well-being that expands on the elements comprising eudaimonia (e.g., purpose in life, autonomy, positive relationships, etc.). On the other hand, Diener et al.’s theory of Subjective Well-Being (1999) captures cognitive and affective evaluations—elements related to hedonia and feeling good. More recently, however, there has been a shift towards understanding well-being by combining elements of hedonia and eudaimonia in one model. For instance, Seligman’s (2011, 2018) PERMA framework of well-being includes elements that comprise both hedonia (e.g., positive emotions) and eudaimonia (e.g., meaning, accomplishment, etc.). As an example, a person who experiences a high sense of meaning (eudaimonia) through a volunteering experience might also be experiencing negative emotions (hedonia) at the time of volunteering at a hospital due to exposure to other people’s ill-being. Assessing this person’s well-being through a framework merely focused on eudaimonia will provide a different assessment of how they are doing compared to an assessment through a framework solely focused on hedonia. Thus, having a picture of both hedonia and eudaimonia simultaneously provides more nuances to our understanding of well-being.

In sum, theoretical perspectives on what entails a “good life” have grown exponentially in the past two decades (Rombaoa & Heshmati, 2023). We believe that no one theory can be deemed as the “superior” framework in explaining psychological well-being. Rather, clarifications need to be made around how specific theoretical approaches to well-being may be differentially advantageous for operationalizing this multiplex construct as it relates to people’s lives. A first step towards this clarification is identifying the utility of these theoretically driven operationalizations of well-being as it applies to people’s real-world experiences. Whereas it is important to understand what makes up well-being, it is equally important to recognize the nature of change in these elements as they unfold in daily lives; while some elements may be more stable in the way they are experienced in people’s lives, some others may be more susceptible to change and be state-like in nature rather than trait-like. Hence, assessments of well-being need to be in-line with the types of questions being asked with regards to the good life: Do we want to understand people’s overall sense of well-being (e.g., a nation’s general quality of life), or do we want to understand the day-to-day experiences of living a high quality life (e.g., fluctuations in experiences of positive emotions in their workplace).

## Global Assessments of Well-Being

Global assessments of well-being have been a focus of research for decades, and have become more prevalent with the advent of the field of Positive Psychology (Seligman & Csikszentmihalyi, 2000). These assessments have been shown to be useful for both researchers and clinicians in understanding the degree of an individual's overall quality of life and ways to increase people’s general sense of well-being. World leaders and governments have also shown interest in these assessments to monitor their citizen’s quality of life (Diener, et al., 2009; Stiglitz, et al., 2009). Well-being global assessments are typically focused on the totality of one’s positive experiences in life that requires retrospection via self-reports in survey-based measures.

Despite the ease that comes with answering seemingly straightforward questions, the emphasis on global assessments introduces some limitations in measurements of well-being. First, the reliance on retrospective recall in global measures, introduces unreliable and biased responses; for instance, people are more prone to remember negative events and feelings in their lives when in a negative state (Clark & Teasdale, 1982). Second, global assessments prevent the examination of people’s dynamic moment-to-moment or day-to-day changes in feelings and experiences as a sequence of events over the course of time. Finally, the contextual governance of experiences is neglected in global measures, eliminating an in-depth understanding of how well-being varies in the context of others or in interaction with the environment as life is lived in real-time and real-world settings (Shiffman et al., 2008). These limitations have cast some doubt in the validity of global self-report measures (Hudson et al., 2020; Kahneman, 1999; Schwarz & Strack, 1999). Experiences of well-being, however, unfold in a momentary manner in people’s daily lives in their naturalistic context. Thus, examinations of people’s experiences of well-being as they live their lives day-to-day and moment-to-moment in real-world contexts, is imperative to a more precise understanding of well-being.

## Momentary PERMA (*m*PERMA)

Capturing well-being in real-time and real-world contexts is an important new direction to further our understanding of PERMA. People’s well-being undergoes dynamic changes governed by their contextual living circumstances and interactions with their immediate and extended environment as it unfolds over a sequence of events over both short and longer time scales (Donaldson et al., 2021). It is these momentary experiences that accumulate to generate global evaluations of one’s well-being (Heshmati et al., 2022). As a result, our understanding of the dynamic changes in well-being and its indicators over time and across contexts are lost when we use merely global assessments of well-being. By using experience sampling methods (Csikszentmihalyi & Larson, 1987) such as Ecological Momentary Assessment (EMA; Stone & Shiffman, 1994) we can move beyond the limitations of global assessments and take a dynamical modeling approach to measure how indicators of well-being *change* in people’s momentary and daily experiences.

Specifically, we can recognize patterns in people’s moment-to-moment experiences of elements of well-being and examine how those varying patterns of individual differences relate to more long-term and stable reports of well-being. For instance, when using momentary reports of feeling loved – a measure of one of the PERMA elements, positive relationships – over short time-scales, we can examine love in terms of (a) mean levels: a person’s average levels of felt love across time, as well as (b) intra-individual variability (IIV): how much a person’s levels of feeling loved fluctuate around their mean, over day-to-day momentary experiences. With this, we can spot time-structured change patterns that amount to how much a person’s experience of positivity in their day-to-day relationships is related to longer term experiences of well-being (Oravecz et al., 2020).

Thus far, using experience sampling methods, researchers have made great advancements in the study of affect–an important component of subjective well-being. Through investigations of within-person change in positive and negative emotions in shorter time scales and its manifestation as a momentary state in daily life, research has demonstrated links between mood variability and more stable states and traits such as depressive states (McConville & Cooper, 1996), psychological health (Gruber et al., 2013; Goicoechea et al., 2023), physical health (Hardy & Segerstrom, 2017), emotional eating (Heshmati et al., 2023), lifestyle (Rombaoa et al., 2023); and age (Rocke & Brose, 2013; Larson et al., 1980). In fact, in a study conducted by Eid and Diener (1999), the validity of hedonic variability, as measured by positive and negative affect IIV across time, was examined in relation to average hedonic levels and personality traits. Eid and Diener (1999) found that hedonic variability, especially variability in positive emotions, is “sufficiently stable [and distinct] to be considered a psychological trait” (Eid & Diener, 1999; p. 662).

Whereas examining within-person characteristics of momentary changes of affect has provided insight into hedonic well-being, less is known regarding within-person changes of eudaimonic elements of well-being. Moreover, within-person dynamics in both hedonia and eudaimonia, as a multidimensional framework, has not been examined in relation to more stable and global reports of well-being. For this purpose, we developed a momentary measure of PERMA (*m*PERMA)–as a momentary and multidimensional measure of well-being– by adapting the PERMA-Profiler (Butler & Kern, 2016) items for the Ecological Momentary Assessment paradigm (EMA; Stone & Shiffman, 1994).

The process we adopted in developing a state-measure of PERMA (i.e., *m*PERMA) was in line with the suggested procedures by Horstmann and Ziegler (2020). Horstmann and Ziegler (2020) suggest that for constructing measures suitable for experience sampling methods (i.e., state measures), researchers need to answer three questions that inform item construction and scale development. First, we needed to consider what is the construct that is being measured*.* We aimed at measuring both hedonic and eudaimonic elements that make up well-being. The PERMA theory (Seligman, 2011, 2018) incorporates both aspects and has a validated trait-level measurement instrument, the PERMA-Profiler (Butler & Kern, 2016), therefore we chose this tool as a basis for ours.

Second, we considered the intended purpose of the measurement. Our goal was to capture experiences of well-being in people’s daily lives in real-world contexts and in-situ. The PERMA-Profiler validated three items per PERMA element for measuring PERMA as a trait (Butler & Kern, 2016), which can be considered parsimonious given the complexity of PERMA. To make them appropriate for momentary assessments where participants respond to items multiple times per day, we adapted the phrasing for reflecting daily life states of well-being. That is to say that *m*PERMA adopts the dimensionality of the PERMA-Profiler scale to capture the five PERMA elements, but adapts the trait-level language of the PERMA-Profiler items (e.g., “In general, how often do you feel joyful?”) to momentary based assessments of well-being (e.g., “How joyful do you feel right now?”). Additionally, the measure should include items measuring each element of well-being appropriate to different situations of a person’s life. For instance to capture Engagement, we have situational-specific items such as “When I noticed the text message, I was absorbed in what I was doing” or “When I noticed the text message, I felt excited and interested in the things around me.”

Third, we considered the targeted population*.* The *m*PERMA measure is targeted for the general population, specifically adults 18 years and older. There has been a plethora of research studies focusing on different elements of well-being in adults’ lives, both on the trait and state level. Specifically, PERMA-Profiler was originally validated using an adult population (Butler & Kern, 2016) and subsequent research validating this measure with participants 18 years and older (e.g., Bartholomaeus et al., 2020; Donaldson et al., 2021; Umucu et al., 2020) and tested globally (e.g., Giangrasso, 2021; Ryan et al., 2019; Wammerl et al., 2019). However, it is essential to acknowledge that the sample used in the current study for the purpose of validation of the measurement was limited to college students 18–22 years of age. This decision was primarily driven by the time-intensive nature of our measurements and the convenience of using a college student sample which is often more easily accessible for research involving detailed measurement designs. We elaborate on this further in the limitations section of this paper.

## The Current Study

In the current study, we first aimed to test the factor structure of *m*PERMA, a momentary scale developed to measure the PERMA building blocks in real-time and real-world contexts. After validation of the measure, we examined how within-person dynamics of each of the PERMA building blocks—measured via *m*PERMA—were associated with global measures of SWB, the Flourishing Scale (FS; Diener et al., 2009) and the Emotional Well-Being subscale of the Short Form Health Survey (SF-36; Ware & Sherbourne, 1992). We selected the FS as our global assessment of psychological well-being because through a single well-being score it assesses various self-perceived elements of well-being (e.g., relationships, self-esteem, purpose, etc.) from a global perspective and has been shown to be strongly associated with other global well-being scales (see Diener et al., 2009). Additionally, the FS is proposed to be aligned with advancements in the psychological theories of human flourishing that have emerged in recent years. The authors have incorporated insights from multiple contemporary theories, including those proposed by Ryff(1989), Ryff & Singer (1998), and Ryan and Deci (2000). These theories emphasize the importance of universal human psychological needs, such as competence, relatedness, and self-acceptance, which the FS is proposed to effectively capture (Diener et al., 2009). However, it should be noted that FS was not shown to be associated with autonomy, a component of eudaimonic well-being present in Ryff’s (1989) Psychological Well-Being framework and hence this element is missing from our examination of eudaimonic well-being. In addition to the FS that captures the global *eudaimonic* aspects of well-being, we used the Emotional Well-Being subscale of the SF-36 to capture the global *hedonic* dimensions of well-being.

Specifically, we examined *m*PERMA in terms of mean levels and its dynamics of IIV. Previous research examining the dynamical characteristics of well-being elements demonstrated that IIV in these elements (e.g., positive emotions; Eid & Diener, 1999) are sufficiently distinct to be considered as unique traits. We used this dynamical characteristic of *m*PERMA (i.e., IIV) to explore whether there are systematic associations between this and more stable well-being measures. Specifically, we tested the association of mean levels and fluctuations (IIV) in each PERMA element in relation to globally assessed levels of hedonic (i.e., emotional well-being) and eudaimonic (i.e., Flourishing) well-being.

Previous reports of within-person dynamic correlates with physical health and mental health indicators have shown that higher variability in affect is negatively associated with health indicators both short-term and long-term, while higher mean levels of positive affect is related to better mental health (e.g., Hardy & Segerstrom, 2017; Ram & Gerstorf, 2009). Moreover, a study examining intra-individual variability in components of subjective well-being (e.g., life satisfaction, positive emotions, negative emotions) in relation to global assessments of psychological states (e.g., social support) found that higher IIV is negatively correlated to social support and positively associated with adverse life events (Gadermann & Zumbo, 2007). A more recent study examining momentary experiences of love in everyday life in relation to more global assessments of psychological well-being (e.g., flourishing, emotional well-being, gratitude) found that higher mean levels of momentary feelings of love were positively associated with psychological well-being (Oravecz, et al., 2020). Thus, we hypothesized that higher mean levels and lower fluctuations (IIV) in the momentary experiences of the Positive emotions element of PERMA would be associated with higher levels of emotional well-being. Similarly, we expected that higher mean levels and lower IIV in the other four elements of the PERMA building blocks (i.e., E, R, M, A) would be associated with higher levels of Flourishing. By articulating building blocks of well-being in terms of dynamic characteristics we can identify various ways through which well-being may be manifested and is experienced in a day-to-day manner that is related to and may lead to higher levels of global well-being.

## Method

In this section, we report how we determined our sample size, all data exclusions (if any), and all measures in the study. The data collected in this study and the respective analytic codes are publicly available and can be accessed through the Open Science Framework (OSF; ​​https://osf.io/emyzp/?view_only=3370dc87c5914b85870a83ea22e3b53b).

## Participants

Participants consisted of a total of 160 undergraduate students at a major public university in the North East, United States. Participants were recruited by a convenience sampling method through the university’s research website. Sixty-eight percent of the participants were female and 32% of the participants were male. Participants’ age ranged from 18 to 22 years old. Seventy-four percent of the participants were White/Caucasian, 6% of them were Black/African-American, 9% of them were Asian or Pacific Islander, and 4% of them were Hispanic/Latino. The remaining 1% identified themselves as other races. This project has been approved by The [blinded] Human Subjects Protection Program and IRB protocol number: STUDY00006362. Informed consent was obtained from all participants before they were involved in the study.

## Study Design

This study is part of a larger study which includes a 56-day intervention study using a signal-contingent EMA design. In the current study we used the EMA data associated with the first 14 days of the larger study, the period before the intervention began. The aim of the study was voiced as measuring psychological well-being in everyday life while also completing some small exercises. On the first day of the study, participants completed an introductory survey on the online Qualtrics survey platform (Qualtrics, Provo, UT) which included the Flourishing scale, Short Form Health Survey, demographics questions, and other psychological scales on a computer in the lab. Starting from the next day, participants received the EMA surveys via text messages to their smartphones six times a day, semi-randomly during the day, for 14 days. The semi-random schedule of the text messages was in a way that surveys were sent to participants’ phones randomly within six designated two-hour blocks across the day with no less than half an hour between each survey. In these surveys, participants gave structured self-reports on their momentary well-being (momentary PERMA). The surveys were scheduled based on the participants’ waking hours, between the time in the morning that they reported waking up until the time they reported they usually went to bed. To reduce the participant workload in the momentary assessments, two items out of the three items for each element were randomly selected and implemented within each momentary assessment and one item was kept missing systematically using the random missingness paradigm. Therefore, in each session participants answered 10 items (2 × 5). As missing data comes from the design, the missingness mechanism is considered completely at random (MCAR, Rubin, 1976). We used maximum likelihood methods to impute the missing data as it produces unbiased estimates of population parameters (Davey, 2009; Enders, 2010; McKnight et al., 2007). Participants were prompted to respond to EMA surveys 6 times a day totaling to 84 survey prompts. On average, participants responded to 75 of the 84 prompts (~ 89% compliance rate). participants were incentivised with payments for their time based on the number of surveys they completed by the end of the study duration.

## Measures^1^

### mPERMA

*m*PERMA items were adapted for EMA data collection from the PERMA- profiler scale (Butler & Kern, 2016). To this end, the 15 items from the PERMA Profiler, three items corresponding to each PERMA element, were reworded for the present moment replacing the “general” wording of the items. For example, the PERMA profiler item “In general, how often do you feel joyful?” was converted to the statement “I am feeling joyful” with response options ranging from “Not at all” to “Extremely” using a sliding scale of 0 to 100. The Cronbach’s alpha coefficients were 0.88 for positive emotions, 0.85 for engagement, 0.91 for relationship, 0.92 for meaning and 0.92 for accomplishment.

### Flourishing

We used the Flourishing scale (Diener et. al., 2009) to measure participants' global assessments of well-being. This scale has eight items corresponding to one’s own perception of success in important domains of well-being such as relationships, purpose, self-esteem, and optimism. Example items of this scale include: “I lead a purposeful and meaningful life.” and “I am optimistic about my future.” The final scale score was calculated over eight items that ranged between 1 (Strongly disagree) to 7 (Strongly agree) where higher scores correspond to better well-being. The Cronbach’s alpha coefficient for this scale was 0.93 indicating high reliability. Overall, participants’ average flourishing score was 5.86 (*SD* = 1.11).

### Emotional Well-Being

The emotional well-being subscale of the 36-item short-form health survey (SF-36; Ware & Sherbourne, 1992) was used to provide a hedonic global assessment of well-being. This subscale included five items around people’s emotional experiences with the prompt “These questions are about how you feel and how things have been with you during the past 4 weeks. For each question, please give the one answer that comes closest to the way you have been feeling.” Items began with “How much of the time in the past 4 weeks…” with example items such as “have you felt downhearted and blue?” or “have you felt calm and peace?” or “have you been a happy person?” with a response scale ranging from 1 (All of the time) to 6 (None of the time). After reverse coding some items, we rescaled the responses to 0–100 to match other measures. Final scores were calculated by aggregating across the five items to form one score representing participants’ emotional well-being score with higher scores indicating higher emotional well-being. This scale demonstrated substantial reliability with a Cronbach’s alpha of 0.82. Overall, participants’ average emotional well-being score was 71.79 (*SD* = 16.64).

## Data Analysis

The sample size was determined based on the larger intervention study with recruiting sufficient samples for each of the three experimental groups. We tried to adhere to general sample size guidelines for conducting factor analysis in the multilevel framework (Comrey & Lee, 1992; Kline, 1994; Maas & Hox, 2005). From the recruited sample of 165, 5 were excluded due to incomplete data (below 80% completion of all surveys) and participant attrition. The analysis was conducted on the final sample of *N* = 160 with a total of 10,979 observations.

The data analysis for this study involved two steps. First, multilevel factor analysis (MFA) was conducted to validate the five-factor structure of *m*PERMA. The multilevel approach was chosen due to the nested nature of the EMA measurements that were clustered within-person. Second, a Bayesian regression analysis was conducted to investigate the association between global levels of flourishing and within-person means and the dynamical characteristics of *m*PERMA elements such as fluctuations of the five *m*PERMA elements. The factor analysis was conducted in the R statistical software (Version 4.2.2., R Core Team, 2022). Specifically, the lavaan package (Rosseel, 2012) was used for MFA, and the psych (Revelle, 2021), stats (Bolar, 2019), and ggplot (Wickham et. al, 2016) packages were used for descriptive statistics. JASP (JASP Team, 2022) was used to conduct the Bayesian regression analysis.

### Multilevel Factor Analysis

To address the nested nature of our data and to be able to consider both levels simultaneously, we conducted a Multilevel Factor Analysis (MFA). Traditional single level Confirmatory Factor Analysis (CFA) uses the total covariance matrix of within-person indicators pooled across occasions, time points, or groups. MFA, on the other hand, decomposes the total covariance matrix into two covariance matrices: within-level (S_pw_) and between-level (S_b_) and enables us to test factor structures at each level simultaneously.

In the current study, the within-level covariance matrix corresponds to the within-person indicators pooled across different time points, and between-level covariance matrix corresponds to the person-level indicators (aggregated over individuals). We hypothesized that at both within- and between- levels, the five-factor PERMA structure would be preserved.

We followed Muthen’s (1994) five-step procedure and defined the models in each step based on Huang (2017). These steps are mainly hierarchical and require some degree of model fit or misfit at each step to continue to the following steps. These steps are briefly explained below. Detailed information about the steps and corresponding models can be found in Huang (2017).

*Step 1:* The first step is fitting a single level (level 1) conventional CFA model using within-level covariance matrix (S_pw_). This step enables researchers to test different factor structures under the theory. Although the nested nature of the data may cause biased parameter estimates, an acceptable model fit is required to follow the next steps. We tested the 5-factor PERMA model in this step.

*Step 2: A null model* is defined and tested in the second step. The null model includes within-level (S_pw_) and between-level (S_b_) covariance matrices in a multigroup setting and the model defined in Step 1 is tested with all equality constraints set to be equal. The poor model fit in this step implies that there is a need for multilevel modeling.

*Step 3:* This step requires fitting an *independence model*. In this step we start to model between-level factor structure by creating group-level factors for each item. These group-level factors are estimated only for the between-level and must be scaled to map onto observed between-level variance. This is achieved by fixing each group-level factor to a scaling factor (approximately *N/g* for balanced groups). This scaling factor is used to scale the biased estimator using the data’s between-level variance so we can estimate the population’s between-level covariance matrix. The population covariance matrix is expected to be comprised of 1 unit of within-group variance and the scaling factor number of units of the between-group variance. Poor model fit in this level indicates that there is a between level variance that needs to be modeled, leading to the next step.

*Step 4:* In this step, *a saturated model* is tested. A saturated model includes the model defined in step 3 and expands that model by allowing covariations in between level factors. One should expect an acceptable model fit similar to step 1 in this step.

*Step 5:* The final step requires modeling the between-level factor structure as hypothesized. Our *hypothesized model* is the five-factor PERMA model both within and between levels.

Conventional fit indices were used in our model fit evaluation: root-mean-square error of approximation (RMSEA), and standardized root-mean-square residual (SRMR) comparative fit index (CFI), and Tucker-Lewis Index (TLI). Hu and Bentler’s (1999) cut off values were considered (CFI and TLI ≥ 0.95; RMSEA and SRMR ≤ 0.08). Moreover, level-specific reliability values were calculated for the final model.

### Calculating Intra-individual Characteristics of mPERMA

The intra-individual characteristics of interest for the current study were (1) within-person mean levels and (2) IIV in the *m*PERMA elements.

## Mean levels

**Fig. 1:**
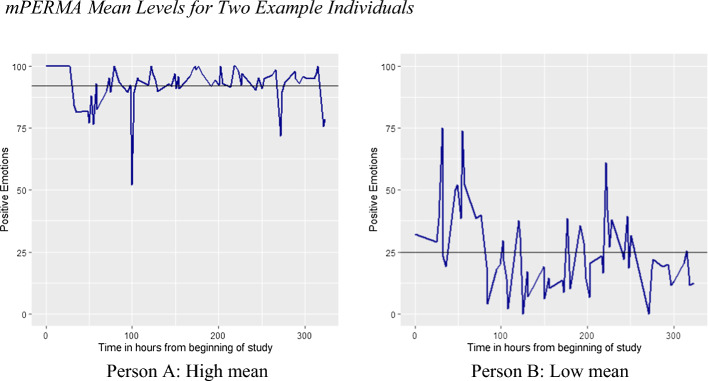
*mPERMA Mean Levels for Two Example Individuals Note.* Each plot demonstrates levels of positive emotions reported by a single person 6 times a day for the duration of 14 days of the study. The straight dark black line illustrates the mean level of their positive emotions. Person A (left plot) demonstrates high mean levels and Person B (right plot) demonstrates lower mean levels in their reported positive emotions

For each *m*PERMA element, we calculated a mean value for every individual separately. Mean levels correspond to the mean value of each individual’s scores over measurements across time. Figure 1 illustrates two individuals' *m*PERMA scores over time. The straight black line indicates the mean level of each example person’s data across time. The plot on the left demonstrates person A with a high mean score in positive emotions across the study time, and the plot on the right shows Person B with a low mean score in their positive emotions.

## Intra-Individual Variability (IIV)

**Fig. 2:**
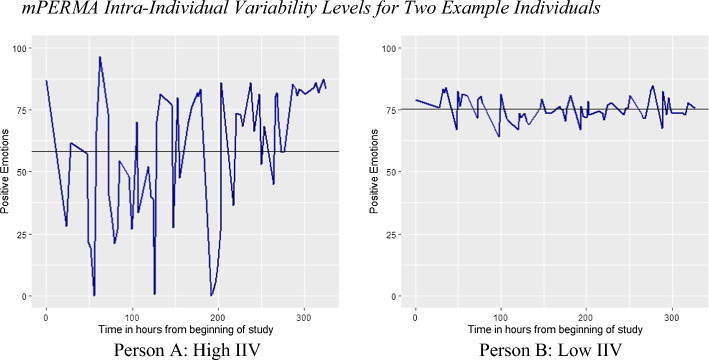
*mPERMA Intra-Individual Variability Levels for Two Example Individuals Note.* Each plot demonstrates levels of positive emotions reported by a single person 6 times a day for the duration of 14 days of the study. The straight dark black line illustrates the mean level of their positive emotions and the blue fluctuating line illustrates the amount of variation in positive emotions across moments and days. Person A (left plot) demonstrates high intra-individual variation levels and Person B (right plot) demonstrates lower intra-individual variation levels in their reported positive emotions

Besides mean levels, we also calculated people’s fluctuations around the mean (IIV) individually for each person. The IIV values correspond to standard deviations of each person’s scores over measurements across the study duration. Figure 2 shows two example individuals who demonstrate different patterns in IIVs for the Positive emotions element of *m*PERMA: Person A (left plot) demonstrates high variability and person B (right plot) shows low variability respectively. Note that although mean levels (straight black line) of both people are pretty similar, their experiences of positive emotions fluctuate differently over time.

### Bayesian Regression Analysis

We conducted Bayesian regression analysis (see, e.g., Wagenmakers et al., 2017; Gelman et al., 2013) to study whether an individual’s general levels of flourishing is related to mean levels and intraindividual variations in the different *m*PERMA elements. Bayesian linear regression aims to determine the most optimal posterior distribution of model parameters, where the posterior distribution is derived based on the likelihood (data) and prior distribution. As we did not have any assumptions about the prior distribution of the variable, we used default priors in the analysis. When estimating models in the Bayesian framework using JASP, we need to manually specify the prior distributions for each model parameter. Current regression analysis consists of estimating 10 regression coefficients (mean and intraindividual variability of mPERMA factors), the intercept, and residual variance. We utilized the default “uninformative” priors provided in JASP. The Bayesian approach to linear regression is useful in our case as we want to explore the effects of several (ten) predictors simultaneously. The inferential output of the Bayesian regression allows us to compare different regression models corresponding to different numbers and combinations of predictors, and evaluates the most optimal combination in terms of predicting our outcome variable (flourishing). We conducted our analysis in JASP. The files containing the analysis and the data are available at the paper’s OSF site.

## Results

There were a total of 10,979 observations for 160 participants with an average of 75 observations per participant. Descriptive statistics for each item and its corresponding intra-class correlations are reported in Table 1. As shown in Table 1, the ICC values are between 0.20 and 0.62 indicating a considerable amount of between-level variance among individuals. Typically, the norm for state measures is to show ICCs ranging from 0.20 to 0.50 (Podsakoff et al., 2019; Sherman et al., 2015; Sun & Vazire, 2019). Variations in the ICCs demonstrated in Table 1 indicate that some of the *m*PERMA elements may have better sensitivity to situational or contextual factors (e.g., Engagement and Positive emotions) compared to others with higher ICCs (e.g., Meaning and Relationship) that may be more attributable to the person. Further descriptive statistics for the IIVs and mean levels of each PERMA element is presented in the supplementary material.

**Table 1:** Descriptive Statistics and ICCs for Each mPERMA Measure

Items	*M*	*SD*	ICC
*Positive emotions*			
I am feeling joyful	67.34	22.31	0.364
I am feeling positive	71.91	20.07	0.373
I am feeling contented	71.32	20.36	0.334
*Engagement*			
When I noticed the text message, I was absorbed in what I was doing	63.65	28.30	0.218
When I noticed the text message, I felt excited and interested in the things around me	57.96	28.61	0.200
When I noticed the text message, I had lost track of time because of what I was doing	58.17	29.41	0.218
*Relationships*			
I feel helped and supported by others	74.78	17.95	0.527
I feel loved	74.74	19.34	0.530
I feel satisfied with my personal relationships	74.54	19.37	0.533
*Meaning*			
I lead a purposeful and meaningful life	76.46	17.64	0.602
What I do in my life is valuable and worthwhile	76.04	17.83	0.616
I have a sense of direction in my life	74.72	18.94	0.548
*Accomplishment*			
I am making progress towards accomplishing my goals	73.51	18.61	0.486
I am achieving the important goals I have set for myself	72.86	18.974	0.488
I am handling my responsibilities	71.79	20.17	0.437

## Multilevel Factor Structure of mPERMA

Results from the MFA analysis are summarized in Table 2. As shown in Table 2, the level one CFA model fitted the data properly (RMSEA = 0.017, SRMR = 0.018, CFI = 0.996, TLI = 0.994) indicating that we had a solid within-level factor model, thus we continued to test the between-level variance factor model. The fit statistics for the null model, as expected, showed a poor fit indicating that there was a between-level variance that we need to take into consideration (RMSEA = 0.162, SRMR = 0.695, CFI = 0.548, TLI = 0.526). This encouraged us to fit the independence model to test whether this variance is important enough to define a between-level model. The independence model indicated an acceptable model fit for two of the fit indices (RMSEA and SRMR), and the CFI and TLI fit indices were also close to a good fit (RMSEA = 0.074, SRMR = 0.027, CFI = 0.913, TLI = 0.902). Therefore, for quality assurance, we moved onto the fourth and fifth steps of the MFA modeling to test group level modeling. In the saturated model, we got an acceptable model fit and moved on to test the hypothesized model (RMSEA = 0.024, SRMR = 0.018, CFI = 0.996, TLI = 0.989).

**Table 2:** Overall Model Fit for Each Model Towards a Multilevel Factor Analysis

Model	*X*^2^ (df)	RMSEA	SRMR	CFI	TLI
Level one model	341.571 (80)	0.017	0.018	0.996	0.994
Null Model	29,142.414 (200)	0.162	0.695	0.548	0.526
Independence model	5742.780 (185)	0.074	0.027	0.913	0.902
Saturated model	341.638 (80)	0.024	0.018	0.996	0.989
Hypothesized model	534.707 (160)	0.021	0.018	0.994	0.992
Modified model #1	626.768 (165)	0.023	0.020	0.993	0.991

Model resulted in negative variance estimates in between-model. However, they are not significantly different from zero for α = .01

The hypothesized model included a five-factor structure for both within and between models. Of note, we updated the scaling of our latent factors via manifest items that produced interpretable factor loadings. The results showed that the hypothesized model fits the data well (RMSEA = 0.021, SRMR = 0.021, CFI = 0.994, TLI = 0.992). The standardized factor loadings are reported in Table 3. The factor loadings in both within and between levels were significantly larger than zero (*p* < 0.00). Factor correlations are shown in Table 5. At the within-level, factor correlations range from highest magnitude of 0.498 (Positive emotion with Relationship) to lowest magnitude of 0.129 (Engagement with Meaning). At the between-level, factor correlations range from highest magnitude of − 0.880 (Meaning with Accomplishment) to lowest − 0.302 (Engagement with Meaning).Results indicate that the items “I am feeling positive” (Positive emotion), “I lead a purposeful and meaningful life” (Meaning), and “I am making progress towards accomplishing my goals” (Accomplishment) show standardized factor loadings > 1 (Table 3) and negative between-level residual variances (Table 4). The between-level variance for “I am feeling positive” (*p* = 0.076), and “I lead a purposeful and meaningful life” (*p* = 0.670) were not significantly different from 0, however, “I am making progress towards accomplishing my goals” was (*p* = 0.017). Additional variances that were not significantly different from 0 include “I was absorbed in what I was doing” (Engagement; *p* = 0.699), “I feel helped and supported by others” (Relationship; *p* = 0.497, and “What I do in my life is valuable and worthwhile” (Meaning; *p* = 0.399).

**Table 3:** Standardized Factor Loadings for Hypothesized Model

	Within-Level		Between-level	
	Estimate	*P*( >|z|)	Estimate	*P*( >|z|)
*Positive Emotions*				
Joyful	0.761		0.965	
Positive	0.843	.000	1.006	.000
Contented	0.683	.000	0.931	.000
*Engagement*				
Engagement 1	0.823		0.998	
Engagement 2	0.688	.000	0.940	.000
Engagement 3	0.783	.000	0.978	.000
*Relationship*				
Relationship 1	0.763		0.999	
Relationship 2	0.750	.000	0.986	.000
Relationship 3	0.717	.000	0.975	.000
*Meaning*				
Meaning 1	0.766		1.000	
Meaning 2	0.787	0.000	0.999	.000
Meaning 3	0.591	0.000	0.949	.000
*Accomplishment*				
Accomplishment 1	0.824		1.002	
Accomplishment 2	0.822	.000	0.997	.000
Accomplishment 3	0.741	.000	0.976	.000

Estimated using maximum likelihood in lavaan (1031 iterations)

**Table 4:** Estimated Residual Variances for Hypothesized Model

			Within	Std Err	*p*	Between	Std Err	*p*
*Positive Emotionality*								
P1	Feeling joyful		133.347	2.63	0.000	12.32	1.916	0.000
P2	Feeling positive		72.816	2.057	0.000	− 1.729	0.973	0.076
P3	Feeling contented		155.059	2.589	0.000	19.288	2.52	0.000
*Engagement*								
E1	Absorbed in activity		205.809	5.708	0.000	0.696	1.802	0.699
E2	Felt excited and interested		351.492	6.046	0.000	19.575	3.131	0.000
E3	Lost track of time		264.946	6.032	0.000	8.312	2.263	0.000
*Relationship*								
R1	Helped and supported		64.333	1.374	0.000	0.433	0.637	0.497
R2	Feel loved		77.291	1.584	0.000	5.551	1.033	0.000
R3	Feel satisfied		85.699	1.605	0.000	9.725	1.429	0.000
*Meaning*								
M1	Lead meaningful		52.082	1.22	0.000	− 0.174	0.409	0.67
M2	Life is valuable		48.544	1.245	0.000	0.379	0.449	0.399
M3	Sense of direction		107.528	1.708	0.000	19.5	2.398	0.000
*Accomplishment*								
A1	Making progress		58.259	1.355	0.000	− 0.756	0.315	0.017
A2	Achieving goals		59.689	1.37	0.000	0.973	0.379	0.010
A3	Handing responsibilities	102.473	1.808	0.000	8.171	1.125	0.000	A3

We conducted post hoc analyses to explore the three “Heywood cases” (e.g., estimated negative variances, standardized loadings > 1) and assess additional diagnostics regarding model misspecification (Kolenikov & Bollen, 2012). There were three estimated residual variances that were negative (Table 4): for items P2 (− 1.729, *p* = 0.076), M1 (− 0.174, *p* = 0.670) and A1 (− 0.756, *p* = 0.017). The first two were not significantly different from zero, however, the estimated residual variance for the third item (A1) was significant (Table 5). It is not realistic to have negative variances in the population, thus, there is a need to explore and identify causes. These Heywood cases often serve as error indicators to conduct deeper investigations into the parameter estimation process, data quality, model specification, etc. Common causes include outliers, small samples, or model misspecification (Bentler & Chou, 1987; Chen et al., 2001; Kolenikov & Bollen, 2012). Practical guidelines suggest that some Heywood cases are not problematic after conducting diagnostic assessment (Bentler & Chou, 1987). Our data has a large sample to parameter ratio (greater than rule of thumb guidelines of 5 to 1; Bentler & Chou, 1987). To assess model specification, we conducted post hoc analyses with alternative model structures to assess factor structure at the between and within levels (see Table 6).

**Table 5:** Factor Correlation Matrices from the Hypothesized Model

Within-level					
	P	E	R	M	A
*Positive Emotions*					
Engagement	0.269				
Relationship	0.498	0.173			
Meaning	0.390	0.129	0.408		
Accomplishment	0.373	0.161	0.346	0.416	

Between-level					
	P	E	R	M	A
*Positive Emotions*					
Engagement	0.357				
Relationship	0.864	0.316			
Meaning	0.718	0.308	0.797		
Accomplishment	0.773	0.329	0.740	0.838

**Table 6:** *Overall Model Fit for *Post Hoc* Exploratory Analysis of Between-Level*

Model	*X*^2^ (df)	RMSEA	SRMR (between)	CFI	TLI
1-Factor	3128.623 (170)	0.040	0.018 (0.172)	0.954	0.943
2-Factor	2498.327 (169)	0.050	0.019 (0.154)	0.964	0.955
3-Factor	1661.282 (164)	0.041	0.019 (0.160)	0.977	0.970
4-Factor	910.817 (164)	0.029	0.018 (0.139)	0.988	0.985
5-Factor	534.686 (160)	0.021	0.018 (0.093)	0.994	0.992

We conducted a post hoc multilevel exploratory factor analysis to identify the factor structure at both levels. First, we fit a modified model with the non-significant residual variances in the hypothesized model fixed to 0. Model fit does not appear to be improving with these modifications. We conducted post hoc exploratory analysis to assess the factor structure on the between-level with 5-factor structure at the within-level. Huang (2017) suggests that higher order factor structures may be simpler or different from within-level factor structures, thus, we compared model fit across increasing factor structures (See Table 6). Exploratory factor analysis of the total covariance matrix guided measurement model specifications in the exploratory MFA. Overall, the factor correlations are higher in the between-level than the within-level, however, the 5-factor solution indicates better fit looking at the model fit indices and indicates lowest SRMR at the between-covariance level (SRMR_between_ = 0.093). Post hoc explorations indicate that a 5-factor structure is preserved in both the within- and between-levels.

### Reliability

Reliability is reported for each factor at the within and between levels of the model. Reported above in Table 1, ICC is computed for each mPERMA item to indicate the amount of group-level variability to the total variance (Huang, 2017). Technically, the ICC is computed as $\rho = (\sigma_{B}^{2} + \sigma_{W}^{2} )^{ - 1} \sigma_{B}^{2}$ for each item. The ICC represents the amount of variability attributable to the between-individual level (Huang, 2017). ICC is reported following Koo and Li (2016)’s best practices guidelines for assessing reliability and reporting appropriate ICC form. As this study involves assessing individuals on multiple measurements, reliability tests involve estimating the reliability in variance at the between and within levels. ICC estimates were calculated in R [Version 4.2.2; R Core Team (2022)]. ICC Reliability estimates were based on each repeated measure from each individual, defined as group-level variability of each mPERMA item, using MFA. ICC’s ranged from 0.200 to 0.616, indicating that there is variability to be modeled at the between and within levels. Additional estimates of level-specific reliability measures are computed via omega coefficient values (McDonald, 1999). Therefore, we can conclude that *m*PERMA measures may benefit by modeling in the multilevel framework and have adequate reliability in both levels. MFA results also imply that both aggregated and disaggregated measures have the same factor structure with slightly different factor loadings. Following Geoldhof, Preacher, & Zyphur (2014)’s recommendations for assessing multilevel reliabilities, within and between reliabilities were estimated for each subscale individually. The multilevel alphas (Cronbach, 1951) and omegas (McDonald, 1970) are provided in Table 7.

**Table 7:** Reliability Estimates of Individual Multilevel CFA Models for Each mPERMA Subset

	Within	2.5% CI	2.5% CI	Between	2.5% CI	2.5% CI
*Positive Emotions*						
$\alpha$	0.806			0.98		
ω	0.804	0.797	0.81	0.979	0.973	0.985
*Engagement*						
$\alpha$	0.806			0.806		
ω	0.809	0.803	0.815	0.981	0.975	0.987
*Relationship*						
$\alpha$	0.786			0.99		
ω	0.787	0.78	0.794	0.991	0.988	0.994
Meaning^a^						
$\alpha$	0.747			0.988		
ω	0.750	0.742	0.758	0.989	0.985	0.992
*Accomplishment*						
$\alpha$	0.835			0.995		
ω	0.835	0.83	0.841	0.995	0.993	0.996
General mPERMA						
$\alpha$	0.800			0.964		
ω	0.782	0.776	0.788	0.967	0.959	0.975

$\alpha$ are Cronbach’s alpha coefficients and ω are omega coefficients a. Warning message: Some estimated manifest variances were negative

## mPERMA Dynamic Characteristics in Relation to Flourishing and Emotional Well-Being

We conducted a Bayesian regression analysis with person-specific characteristics (i.e., mean levels and IIV) of each *m*PERMA element once in relation to Flourishing and a second time in relation to Emotional Well-Being. We report the correlations among *m*PERMA person-specific characteristics in relation to Flourishing along with Emotional Well-being in Table 1 of the Supplemental Material.

### mPERMA Dynamics and Flourishing

The results for the model comparisons predicting Flourishing are provided in Table 8. The most optimal model for predicting Flourishing (i.e., eudaimonic well-being) is shown in the first row of Table 8. This model included within-person means of relationship (Relationship iMean), within-person means of meaning (Meaning iMean), and intra-individual variations of accomplishment (Accomplishment iSD). The posterior probability of this best fitting model was P(M|data = 0.212) which was much larger than all other models. This model explained 63% of the variance in Flourishing. As indicated by the Bayes Factor on the model odds (BF _M_ = 354.143), after observing data, the odds in favor of this model increased by a factor of 354.143, which can be considered substantial. The BF_10_ indicates the relative predictive adequacy of each model compared to this most optimal model. The model in the second row of Table 8 [R(iMean) + M(iMean)] has a BF _10_ = 0.206. By taking reciprocals of this BF of the model in the second row (1/0.206 = 4.85), we can see that the observed data are 4.85 less likely under this model, when compared to the most optimal one in the first row [ R(iMean) + M(iMean) + A(iSD)]. Similar comparisons can be made throughout the different alternative models in this table.

**Table 8:** Bayesian Regression Model Comparison—Flourishing

Models	P(M)	P(M|data)	BF _M_	BF _10_	R^2^
R(iMean) + M(iMean) + A(iSD)	7.576e -4	0.212	354.143	1.000	0.627
R(iMean) + M(iMean)	0.002	0.116	64.986	0.206	0.608
R(iMean) + M(iMean) + M(iSD) + A(iSD)	4.329e -4	0.059	145.455	0.490	0.634
P(iMean) + M(iMean)	0.002	0.056	29.430	0.100	0.604
R(iMean) + M(iMean) + A(iMean)	7.576e -4	0.048	65.793	0.224	0.620
R(iMean) + M(iMean) + R(iSD) + A(iSD)	4.329e -4	0.041	97.777	0.336	0.633
R(iMean) + M(iMean) + P(iSD)	7.576e -4	0.023	31.686	0.111	0.616
P(iMean) + R(iMean) + M(iMean) + A(iSD)	4.329e -4	0.021	49.997	0.175	0.629
R(iMean) + M(iMean) + A(iMean) + A(iSD)	4.329e -4	0.020	47.736	0.167	0.629
P(iMean) + M(iMean) + A(iSD)	7.576e -4	0.018	24.294	0.085	0.615

Table displays only a subset of models tested, in the order of most predictive to least predictive. “P” stands for Positive emotions, “E” stands for Engagement, “R” stands for Relationships, “M” stands for Meaning, and “A” stands for Accomplishment; all are elements from the PERMA model. “iMean” is the calculated within-person mean and “iSD” is the calculated intra-individual variability for each of the PERMA elements

Averaged model coefficients for each variable across different models predicting Flourishing are summarized in Tables 9. Averaging enables us to capture the overall effect of variables across all models which provides stronger evidence for or against the null hypothesis. The parameters displayed in the first six columns of Table 9 assist in determining whether to include each existing variable as a predictor in the model or not. The remaining columns on the right-hand side of Table 9 provide us with the coefficients of each variable.

**Table 9:** Model-Averaged Posterior Summary for Linear Regression Coefficients of Flourishing

	95% Credible Interval								
Coefficient	P(incl)	P(excl)	P(incl|data)	P(excl|data)	BF _inclusion_	Mean	SD	Lower	Upper
Intercept	1.000	0.000	1.000	0.000	1.000	46.975	0.288	46.410	47.529
Positive emotions (iMean)	0.500	0.500	0.224	0.776	0.289	0.232	0.556	− 0.005	1.730
Engagement (iMean)	0.500	0.500	0.084	0.916	0.091	− 0.002	0.091	− 0.060	0.105
Relationship (iMean)	0.500	0.500	0.866	0.134	6.472	1.561	0.788	0.000	2.693
Meaning (iMean)	0.500	0.500	0.978	0.022	43.654	2.637	0.742	1.236	4.071
Accomplishment (iMean)	0.500	0.500	0.200	0.800	0.249	0.214	0.579	0.000	2.022
Positive emotions (iSD)	0.500	0.500	0.126	0.874	0.144	− 0.035	0.188	− 0.675	0.035
Engagement (iSD)	0.500	0.500	0.113	0.887	0.127	− 0.029	0.143	− 0.508	0.000
Relationship (iSD)	0.500	0.500	0.141	0.859	0.163	0.057	0.213	− 0.038	0.800
Meaning (iSD)	0.500	0.500	0.177	0.823	0.216	0.108	0.326	− 0.104	1.144
Accomplishment (iSD)	0.500	0.500	0.590	0.410	1.441	− 0.560	0.564	− 1.550	0.006

Variables listed on the left column are the list of predictors in terms of within-person means (iMean) and within-person standard deviations (iSD). The second column with P(incl) indicates the prior inclusion probability and third column P(excl) indicates the prior exclusion probability. P(incl|data) in the fourth column indicates the posterior inclusion probability and similarly the P(excl|data) in the fifth column indicates the posterior exclusion probability. The sixth column demonstrates change from prior to posterior inclusion odds through the inclusion Bayes Factor (BF inclusion)

Based on the resulting inclusion Bayes Factors (BF _inclusion_), which is calculated via dividing the posterior inclusion odds [P(incl|data)] by the prior inclusion odds [P(incl)], we found that there is very strong evidence for including within-person mean levels of Meaning (Meaning iMean) as a predictor in the model (BF _inclusion_ = 43.654). In other words, a BF _inclusion_ of 43.654 indicates that the data have increased prior odds by a factor of 43.654 for including Meaning iMean as a predictor in the model predicting Flourishing. This is considered as very strong evidence (100 > BF _inclusion_ > 30) according to Jeffrey’s (1961) classification scheme for interpretation of evidence strength.

Moreover, inclusion of Relationship iMean (i.e., within-person mean levels of Relationship) as a predictor of Flourishing was also supported by the data. Results indicated that there is evidence for including Relationship iMean with a BF _inclusion_ = 6.472; this means that the data increased prior odds by a factor of 6.472 for including this variable. This is considered as moderate evidence (10 > BF _inclusion_ > 3) for the alternative hypothesis of a positive association being present between mean levels of momentary Relationship and global eudaimonic well-being (i.e., Flourishing).

While intra-individual variations in Accomplishment (Accomplishment iSD) were also included in the most optimal model presented in Table 8, The BF _inclusion_ = 1.441 shown in the posterior summaries of coefficients in Table 9 indicated that the strength of this evidence is small (sometimes labeled as “anecdotal”). Importantly, this association is negative (Mean = -0.560) meaning that higher fluctuations in reports of Accomplishment in people’s daily lives is related to lower levels of Flourishing.

With multiple predictors in the model and considering the intertwined characteristic of well-being, we assessed the best prediction model for Flourishing from the regression analyses. With three variables in this model, we assessed for multicollinearity. We computed the Variance Inflation Factor (VIF) values and tolerance values (Table 10) for each variable to investigate multicollinearity (Belsley et al., 1980). The VIF values were below the threshold (VIFs < 4) indicating low correlations among each predictor variable with other predictor variables. The tolerance values are > 0.2 indicating the percentage of unique variables of each predictor that cannot be captured by the other predictor variables.

**Table 10:** Assessing Collinearity of Predictors of Flourishing

	Tolerance	VIF
Relationship (iMean)	0.379	2.641
Meaning (iMean)	0.365	2.738
Accomplishment (iSD)	0.909	1.101

### mPERMA Dynamics and Emotional Well-Being

The results for the model comparisons predicting Emotional Well-Being are provided in Table 11. The most optimal model for predicting hedonic well-being as measured by Emotional Well-Being is demonstrated in the first row of Table 11 which only included within-person mean levels of Positive emotions. The posterior probability of this model (P(M|data) = 0.437) is much larger than all other models. This model explained 35% of the variance in Emotional Well-Being. The Bayes Factor on the model odds (BF _M_ = 84.595) indicates that after observing data, the odds in favor of the model containing Positive emotions iMean as the independent variable increased by a factor of 84.595. By taking reciprocals of the BF _10_ of the most optimal model and the second-best model in the second row (1/0.489 = 2.044) we find that the observed data are 2.044 times more likely under the model containing P(iMean) as the predictor compared to the model with both P(iMean) + R(iMean). Similar comparisons can be made for the rest of the models with respect to the most optimal model.

**Table 11:** Bayesian Regression Model Comparison—Emotional Well-Being

Models	P(M)	P(M|data)	BF _M_	BF _10_	R^2^
P(iMean)	0.009	0.437	84.595	1.000	0.347
P(iMean) + R(iMean)	0.002	0.048	24.647	0.489	0.359
P(iMean) + E(iMean)	0.002	0.035	18.134	0.365	0.356
P(iMean) + R(iMean) + M(iMean)	7.576e -4	0.025	33.812	0.686	0.377
P(iMean) + E(iSD)	0.002	0.025	12.632	0.257	0.353
P(iMean) + M(iMean)	0.002	0.021	10.788	0.220	0.352
P(iMean) + A(iSD)	0.002	0.019	9.542	0.195	0.351
P(iMean) + R(iSD)	0.002	0.016	7.899	0.162	0.349
P(iMean) + M(iSD)	0.002	0.014	6.988	0.144	0.348
P(iMean) + P(iSD)	0.002	0.012	6.122	0.126	0.347

Table displays only a subset of models in the order of most predictive to least. “P” stands for Positive emotions, “E” stands for Engagement, “R” stands for Relationships, “M” stands for Meaning, and “A” stands for Accomplishment; all are elements from the PERMA model. “iMean” is the calculated within-person mean and “iSD” is the calculated intra-individual variability for each of the PERMA elements

Averaged model coefficients for each variable across different models predicting Emotional Well-Being are summarized in Table 12. Based on the resulting inclusion Bayes Factor for Positive emotion iMean (BF _inclusion_ = 35.892), we found that there is very strong evidence (100 > BF _inclusion_ > 30) for including this variable as a predictor in the model; that is, the data have increased prior odds by a factor of 35.892 for including Positive emotions iMean as a predictor of Emotional Well-Being.

**Table 12:** Model-Averaged Posterior Summary for Linear Regression Coefficients of Emotional Well-Being

	95% Credible Interval								
Coefficient	P(incl)	P(excl)	P(incl|data)	P(excl|data)	BF _inclusion_	Mean	SD	Lower	Upper
Intercept	1.000	0.000	1.000	0.000	1.000	71.975	1.062	70.169	74.008
Positive emotions (iMean)	0.500	0.500	0.973	0.027	35.892	8.580	2.488	3.375	12.424
Engagement (iMean)	0.500	0.500	0.169	0.831	0.204	− 0.255	0.737	− 2.534	0.130
Relationship (iMean)	0.500	0.500	0.314	0.686	0.459	1.652	2.892	− 0.027	7.935
Meaning (iMean)	0.500	0.500	0.257	0.743	0.346	− 1.104	2.277	− 6.631	0.000
Accomplishment (iMean)	0.500	0.500	0.129	0.871	0.149	0.287	1.269	− 0.279	4.913
Positive emotion (iSD)	0.500	0.500	0.108	0.892	0.122	− 0.059	0.644	− 1.210	1.599
Engagement (iSD)	0.500	0.500	0.152	0.848	0.179	0.219	0.706	0.000	2.468
Relationship (iSD)	0.500	0.500	0.104	0.896	0.116	− 0.025	0.509	− 0.786	0.886
Meaning (iSD)	0.500	0.500	0.123	0.877	0.140	− 0.159	0.710	− 2.542	0.025
Accomplishment (iSD)	0.500	0.500	0.139	0.861	0.161	− 0.191	0.739	− 2.071	0.271

On the other hand, based on the inclusion Bayes Factors reported for all the other variables in Table 12, we can conclude that including any other variables in the model predicting Emotional Well-Being in fact decreases the prior odds. For example, data decreases our prior odds for including Engagement iMean by a factor of 1/0.204 = 4.90.

Table 12 also provides point estimates for each predictor’s coefficient as well as the uncertainty via 95% credible intervals. However, it should be noted that estimates are conditional on the underlying model–the estimate of the effect of Positive emotions will be different as a sole independent variable in the model compared to when accompanied by other independent variables. To solve this problem, Bayesian model averaging provides a solution: it provides a weighted average for the 95% credible interval such that each estimate is weighted by the posterior probability of including that independent variable in the model (Faulkenberry & Wagenmakers, 2020; Van den Bergh, et al., 2020). With this, the credible intervals reported in Table 12 account for the uncertainty within and across models. Hence, we can reliably conclude that the coefficient of Positive emotion has a posterior mean of 8.580 indicating that with each increase in average levels of positive emotion experience, people’s levels of Emotional Well-Being increase by an average of 8.580 points. Based on the model averaged credible interval, we can say that this coefficient is 95% probable to be between 3.375 and 12.424.

## Discussion

Although there have been steps made towards better understanding and validating building blocks of well-being on a global level, research on daily experiences of well-being as it unfolds in people’s naturalistic environments has been scarce. The current study aimed to bridge this gap by examining the PERMA building blocks of well-being in people’s day-to-day lives through an EMA design. We followed the steps suggested by Horstmann and Ziegler (2020) to create a measure of PERMA suitable for EMA methods. We dub this new iteration of the scale “*m*PERMA” (momentary PERMA). First, we tested the factor structure of *m*PERMA through MFA. We then used the daily life data captured via *m*PERMA to examine the within-person mean-levels and dynamics of change (e.g., fluctuations) in each PERMA element and their relation to people’s globally assessed hedonic (i.e., Emotional Well-Being) and eudaimonic (i.e., Flourishing) well-being.

As hypothesized, the MFA for *m*PERMA revealed a five-factor structure, at both within- and between-levels, indicating a sound measure of the five PERMA building blocks at a momentary level. Specifically, all three items adapted to EMA assessments measuring each of the five PERMA elements loaded significantly onto the respective PERMA factor. Moreover, *m*PERMA measures indicated adequate reliability in both within and between levels of analysis. In other words, both aggregated and disaggregated measures have the same factor structure with slightly different factor loadings. This finding implies that well-being can be assessed through various elements including both hedonic and eudaimonic aspects of well-being as a momentary experience in daily life. Additionally, considering that the reliability of the full scale is also relatively high [coefficient omega of 0.97 (between) and 0.78 (within), McDonald, 1999]), researchers and practitioners can use either the subscale scores individually or the full scale score as a whole, having flexibility to choose the scoring approach that best suits their research questions and client goals. This approach paves the way for further applied investigations of well-being as is experienced on a momentary basis and in a contextually and ecologically valid manner across different populations and in different life circumstances.

Factor correlations also revealed interesting findings about the relationships between different factors of *m*PERMA. At the within-level, factors were moderately correlated and positively associated. This ranged from the highest magnitude of 0.498 between Positive emotions and Relationships to the weakest magnitude of 0.129 between Engagement and Meaning. In contrast, at the between-level correlations varied in strength, with the strongest correlation observed between Positive Emotion and Relationship (0.864) and the weakest between Meaning and Engagement (0.310). These results suggest that the PERMA factors may have unique relationships with each other depending on the level of analysis, indicating the importance of considering the measurement timescale and item framing when examining the associations between different aspects of well-being.

Moreover, the estimated factor correlations at the between-level demonstrated several interesting pairwise comparisons that shed light on the discriminant validity of the facets. Among the facets demonstrating greater discriminant validity, Engagement consistently emerged as a distinct factor. Specifically, the pairwise correlations between Engagement and Relationship, Engagement and Meaning, and Engagement and Accomplishment indicated relatively lower factor correlations, suggesting a clearer differentiation between these facets. Conversely, facets with lower discriminant validity exhibited higher factor correlations with each other. Notably, the pairwise comparisons of Meaning and Accomplishment, Positive Emotion and Relationship, and Relationship and Meaning demonstrated stronger factor correlations, indicating a greater overlap or shared variance between these facets. In sum, these results suggest that Engagement consistently represents a distinct facet with relatively lower correlations with other facets, supporting its discriminant validity. On the other hand, facets such as Meaning, Accomplishment, Positive Emotion, and Relationship demonstrate somewhat higher correlations, indicating a certain degree of overlap in the variance they capture.

To further our understanding of how different elements of well-being unfold over time in day-to-day life, we then used *m*PERMA to obtain within-person dynamic characteristics of each of the PERMA elements assessed over 14 days and 75 momentary time-points. Using the within-person mean levels and dynamic characteristics (i.e., intra-individual variability) we were able to examine what dynamics of change may be associated with global well-being for each PERMA well-being element. Using a Bayesian regression analysis, we first examined these associations in relation to people’s global reports of their eudaimonic well-being as measured by the Flourishing scale. Next, we examined all five *m*PERMA elements with regards to people’s hedonic well-being as measured by the Emotional Well-Being scale.

As hypothesized, we found that eudaimonic elements of PERMA (e.g., Relationship, Meaning, and Accomplishment), measured on a momentary level, in fact were meaningfully associated with Flourishing—a measure of eudaimonic well-being. Moreover, with regards to hedonic well-being (Emotional Well-Being), the momentary Positive emotion element of PERMA indicated the only meaningful association. This finding provides further evidence that while various elements of well-being can be assessed individually on a momentary basis in people's daily lives and natural environments, they also are meaningfully associated with the corresponding aspect of people’s global reports of well-being. Most importantly, for each of these elements, the dynamic characteristic of change that is related to global reports of well-being is different.

Specifically, we found that in relation to global reports of eudaimonic well-being (i.e., Flourishing), people’s within-person mean levels of Relationship and Meaning are meaningfully and positively associated with Flourishing. That is, regardless of within-person fluctuations, the higher a person’s overall average of moment-to-moment Relationship and Meaning is, the higher their global assessments of Flourishing would be. These results suggest that in order for individuals to experience overall high levels of eudaimonic well-being, it is crucial for them to monitor their daily and momentary experiences of positive relationships (i.e., feeling loved and supported; Heshmati et al., 2019; Heshmati & Donaldson, 2020) and meaning in life (i.e., sense of purpose and value; Cohen & Cairns, 2012; Steger & Kashdan, 2007). It is important to note that maintaining consistently high levels of meaning and positive relationships is key, regardless of the fluctuations in these experiences throughout one’s daily life. This finding is in line with previous research examining love in daily life, with similar results: within-person averages of moments of feeling loved in daily life was significantly associated with psychological well-being (Oravecz et al., 2020); this is while fluctuations in those experiences were not significantly associated with well-being.

On the other hand, our results indicate that it’s the within-person fluctuations in experiences of Accomplishment, rather than mean levels, that is meaningfully associated with Flourishing; more importantly, this association is negative. In other words, higher levels of fluctuation in momentary experiences of accomplishment are related to lower levels of eudaimonic well-being. When contextualizing this finding in people’s daily lives, we can infer that having a steady flow of accomplishment and feelings of success towards one's goals is more relevant for people’s overall sense of eudaimonic well-being than having too many ebbs and flows in feeling accomplished and successful in handling responsibilities and achieving goals. It may be that the high points experienced in this state does not offset the low points and therefore a gradual steady pace is more helpful, regardless of how on average they feel momentarily accomplished. We, however, do caution the reader that these implications are based on correlational associations and examination of causal relationships is needed in future studies.

Additionally, we found that Positive emotion – a hedonic element of PERMA– was the only element that highly mapped onto people’s global reports of hedonic well-being as measured by the Emotional Well-Being scale. Specifically, within-person mean levels of Positive emotion were meaningfully associated with Emotional Well-Being, such that the higher on average people’s momentary experiences of positive emotions, the higher their reports of global emotional well-being would be; this is despite how much fluctuations was existent in their momentary experiences of Positive emotions. Contrary to our expectations, variability in positive emotions in daily life was not meaningfully related to global reports of Emotional Well-Being. Prior research examining intra-individual variability in positive and negative affect in relation to depressive symptoms (McConville & Cooper, 1996) and health (Gruber et al., 2013) have found significant relationships. However, this association might not entirely be true for positive emotions and emotional well-being. Most recently, Oravecz et al., (2020) examined intra-individual characteristics of feeling loved in a momentary manner as it related to emotional well-being. Love was treated as a micromoment of positivity resonance in which people would experience moments of positive emotions with another person. Similar to our findings regarding Positive emotions, Oravecz et al. (2020) also found that out of all the different within-person characteristics of momentary love (e.g., mean levels, IIV, inertia), only within-person mean levels of felt love was meaningfully associated with emotional well-being. While our analytic approach does not allow for causal inference, this finding may suggest that people’s global evaluations of their emotional well-being is more sensitive to the average level of their daily life experiences of those emotions compared to the ebbs and flows. In other words, as long as people experience overall higher levels of positive emotions in their daily life, it doesn't matter that at times they might be low on those emotions. In fact, this might be an indication that when positive emotions are low for people, they are experiencing some level of negative emotions (Russel, 2003) which on its own is an important indicator of well-being. Unfortunately, in the current study, we did not include a measure of negative emotions and therefore cannot confirm this inferred conclusion. We suggest that future studies explore the role of within-person experiences of negative emotions along with positive emotions in relation to global Emotional Well-Being.

Notably, we found no evidence for the relationship between within-person patterns of Engagement in daily life and Flourishing; nor with Emotional Well-Being. Based on previous studies that have also examined Engagement on a momentary basis and its relation to other elements of PERMA (Heshmati et al., 2020), momentary engagement in daily life is not a central element in the network of PERMA. This is particularly true for the specific age group that comprised the participants of this study —early adults in the US. Rather, due to the central role of Positive emotions and Relationship in early adults’ network of PERMA well-being (Heshmati et al., 2020), this finding may suggest that Engagement is indirectly related to individuals’ global well-being. In other words, it may be that increases in Engagement are related to increases in positive emotions which in turn relates to Emotional Well-Being. Similarly, momentary experiences of Engagement may be associated with momentary experiences of positive relationships which in turn relates to Flourishing. Indirect associations among these momentary elements of PERMA warrant future research.

Another possible explanation for this finding is that items representing Engagement in *m*PERMA indicate the lowest intra-class correlations (ICC ~ 0.20) among all other items in the current study. These low ICCs imply that Engagement item scores are most sensitive to situational and contextual factors and less related to person-specific factors, hence the lack of significant association between momentary Engagement measures and more person-specific and trait-like Eudaimonic measures such as Flourishing. Worthy of note, items representing Positive emotions have the next lowest ICC (~ 0.30)– after Engagement items– which display their state-like nature and sensitivity to situations and contexts as well. Although all ICC values for all items were in the normal expected range (0.20 to 0.50) for our purposes, Engagement and Positive emotion items’ ICCs lower values may reflect their transitory states as hedonic elements of well-being. On the other hand, Eudaimonic elements of PERMA such as Relationship, Meaning, and Accomplishment fared on the higher range of ICCs (Relationship ~ 0.50; Meaning ~ 0.60; Accomplishment ~ 0.40). ICC values for state scores that are close to one show that more of their variance could be attributed to person-specific factors and be less sensitive to situational circumstances. Although still in the normal range, higher ICCs for the eudaimonic elements of PERMA (i.e., Relationship, Meaning, and Accomplishment) compared to the hedonic elements, may suggest that these items are more trait-like and eudaimonic experiences are less transitory and prone to change due to circumstances and are more likely to be longstanding.

It should be noted that some items in the *m*PERMA item pool do not explicitly include the “present moment” wording. However, in our opinion, this should not be a reason as to whether participants rated these items based on their momentary experiences on the states measured. We believe that several factors contribute to capturing such momentary evaluations on all items of *m*PERMA. Firstly, the initial instructions for the participants emphasize the importance of responding based on momentary experiences. Secondly, the removal of general wording pertaining to overall life evaluations such as items that begin with “in general…” helps focus participants on their present experiences. Lastly, the nature of the assessments and the frequency of prompts throughout the day ensures that participants provide responses in various temporal contexts. While we understand that specifically for eudaimonic items (e.g., purpose) demonstrate lower iSDs and higher ICCs, we interpret these findings as indicators of the stability and lesser variability of these specific well-being elements. In fact, our expectations regarding the stability of certain PERMA elements due to their inherent nature were supported by these results.

While the present study has afforded opportunities for more contextualized and ecologically valid examinations of well-being, we also want to acknowledge that the current study has its limitations. This study is limited in that we used a college sample of early adults to conduct this EMA study, as conducting these types of intensive data collections (6 times a day for 14 days) through technology is more feasible with a population that is comfortable with having smartphones with them at all times. Moreover, the nature of our analysis was a correlational approach and is limited in making causal inferences. Future studies are warranted to explore the causal associations among *m*PERMA elements in daily life and their effects on global assessments of well-being. Furthermore, our study was only limited to the investigation of direct relationships. We also encourage future research to explore indirect relationships among these constructs, in addition to the direct associations. For instance, the role of momentary experiences of engagement and flow in daily life may be further clarified with investigations of indirect mechanisms through which flow state may be related to overall reports of well-being. Additionally, this study encountered potential Heywood cases (negative residual variances) in the model estimation that can affect the analysis. Future studies should conduct rigorous assessments to diagnose the source of these Heywood cases and impact on analysis (Bentler & Chou, 1987; Kolenikov & Bollen, 2012). This study was a first to examine the dynamics of daily PERMA through intensive data and we hope that it provides the foundation for future research to expand the examination of well-being as it is experienced in daily life in other age groups and to more representative samples.

## Implications

The scientific study of well-being has taken big steps in the past decade with more and more research focused on cross-validation of prevalent well-being measures (Donaldson et al., 2020; Goodman et al., 2018; Joshanloo, 2019). Most of these scientific endeavors, however, have been limited to global assessments of well-being in an attempt to understand people’s feelings and evaluations of their lives. These global assessments require retrospection in self-report measures, introducing unreliable and biased responses. Additionally, global well-being measures lack ecological validity, neglecting life as it is lived in real-time and real-world settings (Shiffman et al., 2008). All in all, while advancements in the understanding of how people think and feel about their lives have been made, less research has focused on how well-being is *experienced* in people’s lives (Dolan et al., 2017). To bridge this gap, this study took a step toward expanding the scientific understanding of well-being by examining how *experiences* of a good life unfold in people’s day-to-day lives and how these experiences relate to global evaluations of well-being. We used Ecological Momentary Assessment, as a study design that surpasses recall biases while also providing ecologically valid information regarding people’s experiences of well-being. Through this design, we were able to provide a snapshot of the within-person mean levels and dynamics of change ( IIV) in various elements of well-being (i.e., Positive Emotions, Engagement, Relationships, Meaning, Accomplishment) in people’s own daily contextual environments.

An important implication of the findings of this study is that when building blocks of well-being are broken down into different measurable elements, those elements can be used to validly measure people’s *daily experiences* of well-being, above and beyond their general thoughts and feelings (i.e., evaluations) of their lives. We can then use such momentary assessments of well-being to explore dynamic patterns of people’s lived experiences of elements of well-being (e.g., within-person fluctuation) and study them in relation to other more stable individual characteristics. These kinds of dynamic investigations are often overlooked but are important because, it may be possible that for a specific well-being element high (low) fluctuations of that experience in daily life is related to high (low) flourishing, regardless of their average levels of well-being over time. For example, in our investigation of daily experiences of well-being in college students, we found that momentary experiences of PERMA elements of well-being display different patterns of change as they unfold over time. Moreover, for each element, different aspects of their dynamical change is related to people’s overall experiences of well-being: while high averages of positive relationships across moments is important for people in relation to their global assessments of well-being, accomplishment, however, shows a different pattern; regardless of average levels, experiencing lower fluctuations is meaningfully related to higher global well-being.

Exploring the dynamic of change in momentary experiences of PERMA well-being has implications for applied research in two ways: (1) When people think about well-being, they are most likely to take a unidimensional approach to experiencing and enhancing well-being. However, our research demonstrates that different elements of well-being (P-E-R-M-A) experienced in daily life are related to different aspects of people’s global evaluations of well-being, which underscores the importance of richness in how well-being is experienced in daily life. (2) When intervention scientists or practitioners prescribe practices for increasing global levels of life satisfaction and well-being in people’s daily lives, they usually focus on the types of activities that merely enhance average levels of various elements of well-being from one time to the next. However, this study highlights the importance of dynamics of change in each element of well-being as experienced in daily life and how those dynamics might be related to people’s overall sense of well-being.

For instance, a most recent meta-analysis on the effectiveness of interventions in enhancing eudaimonic well-being (van Dierendonck & Lam, 2022) found that targeted psychological practices and programs that were theoretically linked to Ryff’s Psychological Well-Being (Ryff, 1989) dimensions can improve people’s eudaimonic well-being. This conclusion was however made based on pre- and post- change scores from intervention studies, with no information on what the dynamics of change looked like for each dimension of psychological well-being. The current study suggests that for various elements of well-being it may be more beneficial if interventions target the appropriate dynamic of change for that element as it relates to global well-being. For example, to increase positive emotions, meaning, and positive relationships in people, interventions should target increasing within-person average levels of these momentary experiences. However, for the accomplishment element, interventions should focus on decreases in extreme fluctuations in these experiences, perhaps by keeping people’s sense of achievement and success at a steady level. Thus, as a next step to this recent meta-analysis on effectiveness of psychological well-being interventions (van Dierendonck & Lam, 2022), future research can examine daily dynamic change as a result of well-being interventions to ultimately inform psychological practices that are most effective in enhancing people’s daily experiences of a good life.

## Conclusion

To our knowledge, this research is the first to study the factor structure of momentary assessments of the PERMA building blocks to be used in EMA studies of well-being, while highlighting the importance of the dynamics of daily experiences of these building blocks in relation to global hedonic and eudaimonic assessments. This study also continues the conversation around the validity of PERMA in relation to other well-being measures, as presented in the *Journal of Positive Psychology* debate between Goodman et al. (2018) and Seligman (2018) followed by Donaldson et al. (2020).

With the advancements that this study and the *m*PERMA measure has made in the scientific study of well-being in ecologically valid ways, we hope that the path is paved for the positive psychology scientific community to supplement global measurements of well-being with more contextualized and experiential examinations of this multiplex construct. With this, we hope that a fuller understanding of well-being as it is lived in everyday life is achieved.

### Supplementary Information

Below is the link to the electronic supplementary material.

Supplementary file1 (DOCX 21 kb)

## Footnotes

1 All measures reported here are a selection of measures from the larger study that were relevant for the current study. The larger study included additional measures related to participants’ health, emotions, mindfulness, and life satisfaction.
