# Supplementary material for: Momentary PERMA: An Adapted Measurement Tool for Studying Well-Being in Daily Life
Source: J Happiness Stud. 2023 Sep 22;24(8):2441–72. doi: 10.1007/s10902-023-00684-w (PMC10730635; doi:10.1007/s10902-023-00684-w)
Supplement: Supplementary file 1 — Supplementary file1 (DOCX 21 kb) [file 10902_2023_684_MOESM1_ESM.docx]

**Supplemental Material**

**Descriptives**

Table 1

*Means, Standard Deviations, and Correlations with Confidence Intervals for iMeans, IIVs of PERMA Components and Emotional Wellbeing and Flourishing Measures*

| Variable | *M* | *SD* | 1 | 2 | 3 | 4 | 5 | 6 | 7 | 8 | 9 | 10 | 11 |
| --- | --- | --- | --- | --- | --- | --- | --- | --- | --- | --- | --- | --- | --- |
|  |  |  |  |  |  |  |  |  |  |  |  |  |  |
| 1. Flourishing | 46.88 | 6.03 |  |  |  |  |  |  |  |  |  |  |  |
|  |  |  |  |  |  |  |  |  |  |  |  |  |  |
| 2. EmotionalWB | 72.26 | 17.03 | .59** |  |  |  |  |  |  |  |  |  |  |
|  |  |  | [.54, .63] |  |  |  |  |  |  |  |  |  |  |
|  |  |  |  |  |  |  |  |  |  |  |  |  |  |
| 3. Pmi | 70.44 | 13.19 | .63** | .55** |  |  |  |  |  |  |  |  |  |
|  |  |  | [.58, .67] | [.50, .59] |  |  |  |  |  |  |  |  |  |
|  |  |  |  |  |  |  |  |  |  |  |  |  |  |
| 4. Emi | 60.06 | 14.28 | .26** | .12** | .38** |  |  |  |  |  |  |  |  |
|  |  |  | [.19, .32] | [.05, .18] | [.32, .43] |  |  |  |  |  |  |  |  |
|  |  |  |  |  |  |  |  |  |  |  |  |  |  |
| 5. Rmi | 74.84 | 14.33 | .67** | .50** | .85** | .33** |  |  |  |  |  |  |  |
|  |  |  | [.63, .71] | [.45, .55] | [.83, .86] | [.26, .39] |  |  |  |  |  |  |  |
|  |  |  |  |  |  |  |  |  |  |  |  |  |  |
| 6. Mmi | 75.85 | 14.59 | .70** | .38** | .73** | .31** | .79** |  |  |  |  |  |  |
|  |  |  | [.66, .73] | [.32, .44] | [.70, .76] | [.25, .37] | [.76, .81] |  |  |  |  |  |  |
|  |  |  |  |  |  |  |  |  |  |  |  |  |  |
| 7. Ami | 72.87 | 13.59 | .68** | .43** | .78** | .35** | .74** | .86** |  |  |  |  |  |
|  |  |  | [.64, .71] | [.37, .48] | [.75, .81] | [.28, .41] | [.71, .77] | [.84, .88] |  |  |  |  |  |
|  |  |  |  |  |  |  |  |  |  |  |  |  |  |
| 8. Psd | 13.58 | 5.10 | -.18** | -.26** | -.39** | -.14** | -.11** | -.16** | -.26** |  |  |  |  |
|  |  |  | [-.25, -.12] | [-.32, -.19] | [-.45, -.33] | [-.20, -.07] | [-.18, -.04] | [-.23, -.09] | [-.32, -.19] |  |  |  |  |
|  |  |  |  |  |  |  |  |  |  |  |  |  |  |
| 9. Esd | 21.21 | 6.90 | .03 | .01 | .05 | -.21** | .19** | .13** | .07* | .52** |  |  |  |
|  |  |  | [-.04, .10] | [-.06, .08] | [-.02, .11] | [-.28, -.14] | [.12, .25] | [.06, .20] | [.01, .14] | [.46, .56] |  |  |  |
|  |  |  |  |  |  |  |  |  |  |  |  |  |  |
| 10. Rsd | 9.97 | 4.17 | -.23** | -.23** | -.34** | -.18** | -.36** | -.29** | -.32** | .59** | .28** |  |  |
|  |  |  | [-.29, -.16] | [-.30, -.17] | [-.40, -.28] | [-.25, -.11] | [-.42, -.30] | [-.35, -.23] | [-.38, -.25] | [.54, .63] | [.22, .35] |  |  |
|  |  |  |  |  |  |  |  |  |  |  |  |  |  |
| 11. Msd | 8.57 | 3.95 | -.30** | -.23** | -.29** | -.09** | -.26** | -.47** | -.47** | .52** | .24** | .65** |  |
|  |  |  | [-.36, -.23] | [-.30, -.17] | [-.35, -.22] | [-.16, -.02] | [-.32, -.19] | [-.52, -.41] | [-.52, -.41] | [.47, .57] | [.17, .30] | [.61, .69] |  |
|  |  |  |  |  |  |  |  |  |  |  |  |  |  |
| 12. Asd | 11.16 | 5.15 | -.34** | -.28** | -.35** | -.14** | -.24** | -.30** | -.51** | .63** | .37** | .54** | .70** |
|  |  |  | [-.40, -.27] | [-.34, -.21] | [-.41, -.29] | [-.21, -.07] | [-.31, -.17] | [-.36, -.24] | [-.56, -.45] | [.59, .67] | [.30, .42] | [.49, .58] | [.66, .73] |
|  |  |  |  |  |  |  |  |  |  |  |  |  |  |

*Note.* *M* and *SD* are used to represent mean and standard deviation, respectively. Values in square brackets indicate the 95% confidence interval for each correlation. The confidence interval is a plausible range of population correlations that could have caused the sample correlation (Cumming, 2014). * indicates *p* < .05. ** indicates *p* < .01.
